# VOLPES: an interactive web-based tool for visualizing and comparing physicochemical properties of biological sequences

**DOI:** 10.1093/nar/gkz407

**Published:** 2019-05-22

**Authors:** Lukas Bartonek, Bojan Zagrovic

**Affiliations:** Department of Structural and Computational Biology, Max Perutz Labs, University of Vienna, Campus Vienna Biocenter 5, 1030 Vienna, Austria

## Abstract

The structure, dynamics and, ultimately, biological function of proteins and nucleic acids are determined by the physicochemical properties of their primary sequences. Such properties are frequently captured via one-dimensional profile plots depicting a given physicochemical variable as a function of sequence position. Hydrophobicity, charge or structural disorder in proteins or nucleobase-density in nucleic acids are routinely visualized in this manner to analyze sequences at a glance. Such visualizations, however, are typically created case-by-case in a purely static manner, employ fixed visualization parameters only and do not enable a quantitative comparison between different sequences. Here, we present VOLPES (volpes.univie.ac.at), a user-friendly web server and the corresponding JavaScript library that enable a fully interactive, multifunctional visualization, analysis and comparison of the physicochemical properties of protein and nucleic-acid sequences, allowing unprecedented insight into biological sequence data and creating a starting point for further in-depth exploration.

## INTRODUCTION

A foundational principle of bioinformatics is that biological function is encoded in and can ultimately be related to the primary sequences of nucleic acids and proteins. While sequences can be analyzed in different abstract ways, they first and foremost refer to the composition of real, physical objects i.e. linear-chain biopolymers. Importantly, the physicochemical properties of biopolymers are frequently visualized via one-dimensional profile plots where each sequence position is associated with a quantitative measure of the property of interest. For example, hydrophobicity profiles are often employed to learn about the structural features and domain boundaries in proteins. In particular, such profiles are routinely used to ascertain the location of transmembrane regions in membrane proteins (1–4). Another common application of profile plots is to visualize the distribution of charge in protein sequences (5–7). Such analyses, for example, have been instrumental in understanding the fundamental principles behind the formation of phase-separated granules in the cell (8,9). Finally, nucleobase-density profiles in DNA and RNA have been used to study different biological phenomena ranging from nucleosome positioning (10–12) to RNA–protein interactions (13–15) to protein localization (16,17).

While many programs or servers output results in the form of profile plots, these tools are usually highly specialized and/or feature static visualization only (2,18,19). Despite the fact that the usage of such tools is mostly exploratory in nature, users are typically unable to change visualization parameters on-the-fly or opt for a different property without extensive recalculation or even having to switch to a different tool altogether. Furthermore, most tools do not enable a comparison between multiple profiles, although such a feature may be critical in a wide range of applications including mutational analyses and domain comparisons or simply to highlight the relationship between different properties of interest. On the other hand, tools featuring more advanced features are not available as web servers but rather as downloadable standalone packages (3,20).

In response to these challenges, we present here VOLPES, a multifunctional web server and a JavaScript library for highly customizable, interactive visualization, analysis and comparison of the physicochemical properties of protein and RNA sequences. As its integral part, the VOLPES server provides direct access to hundreds of published amino-acid and nucleobase physicochemical property scales, many of which were sourced from the AAindex (21,22), facilitating a rapid and multifaceted exploration of the sequences of interest. By treating biomolecular sequences as physicochemical objects, which they invariably are, VOLPES enables detection of biologically meaningful patterns and similarities that may be inaccessible to standard methods of primary sequence analysis and comparison.

## MATERIALS AND METHODS

### Input

The VOLPES server requires only protein or RNA primary sequence data as the initial user input. All further details required for the analysis are already provided by the server itself. The most basic way of inputting sequence information is either in the form of a string of single-letter-encoded monomers (amino acids or nucleobases) without further specifications or in the FASTA format, which is automatically detected and handled appropriately by the server, including support for gap characters. However, the most convenient way to import sequence data is via an integrated connection to public databases whereby the relevant sequence data and sequence names, if available, are fetched automatically. In the current release, UniProt (23) and ENA (European Nucleotide Archive) (24) identifiers are supported.

#### Savefiles

Input data can also be provided by uploading a previous savefile generated by the server itself or a compatible external program (please see below for details).

### Output

A multi-tiered approach allows users to generate output files with different levels of complexity.

#### Image output

Conveniently available from the export menu, the active visualization state can be exported as a rasterized PNG image. The saved state is exactly the same as that defined by the user while exploring the data and includes all set visualization parameters as well as the zoom and the shift state of the plotting window. Vector data are supported in the format of SVG files, which can be used as a basis for making publication quality figures in post-processing. This allows figures to be easily combined to make more advanced illustrations, adjust font properties and modify colors afterward. Furthermore, this format allows figures to be rendered to rasterized formats at arbitrary resolution.

#### Data output

VOLPES can also be employed to compute physicochemical profile data for uses other than visualization. To accommodate this, output of raw data in the form of CSV files is supported. Users can choose any combination of staged sequence data, including a subset of select profiles, to be downloaded for further use.

#### Savefiles

Any given visualization state can also be exported as a *.viz* savefile to be shared with collaborators or saved for later analysis. These files specify all details about the current visualization visible to the user and, therefore, allow anyone with access to this file to continue working at the same point at a later time. This file standard is open, easy to read/write and allows developers of other tools to access the stored information with ease.

### Interactivity

**Figure 1.**
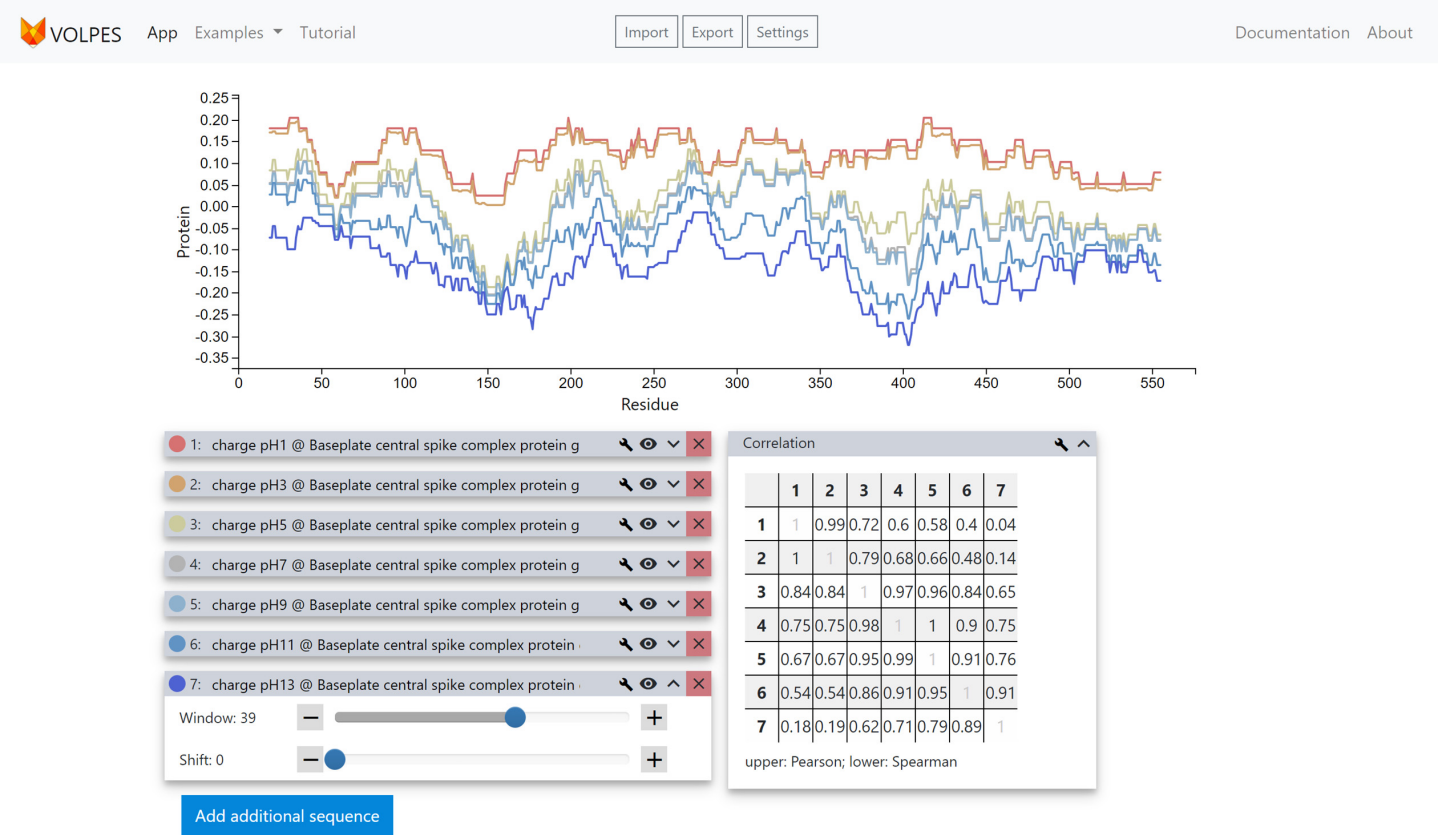
Example of a typical analysis performed via the VOLPES server: charge profiles calculated at different values of pH for a specified protein. A quantitative analysis of the similarity between profiles is given in the correlation matrix.

The principal advantage of VOLPES as compared to other tools for visualizing sequence profiles is its inherent interactivity and ease of accessing the underlying data. This is achieved by performing most of the visualization-related calculations client-side in the users’ browser. In other words, VOLPES enables real-time interactive visualizations by removing any delays introduced by internet connectivity, while all of its modifiable parameters are accessible via an user-friendly interface, as illustrated in Figure 1.

#### Smoothing

Virtually all visualizations of physicochemical properties in the form of profiles employ smoothing of the raw data to make key features stand out, while reducing the inherent noise in the data. The size of the window used for such smoothing depends strongly on the feature of interest. The VOLPES interface allows users to change the smoothing window size by using a slider and observing the impact in real time. This is essential for users to develop an intimate feel for the underlying data.

#### Shifting and zooming

Unlike other related tools, VOLPES allows multiple sequence profiles to be visualized at the same time as well as shifted against each other along the residue axis. This is implemented on an individual sequence basis such that each sequence can be shifted against all others independently. To keep the drawing canvas to a reasonable size, the implementation ensures that shifts are limited in a way that all possible relative superpositions are possible, but not more. Importantly, users can visualize each profile in relative units of the standard deviation of the corresponding data series, thus enabling superposition of profiles that capture different physicochemical variables. At last, users can zoom in or zoom out of any region in a given profile at will.

#### Visual appearance

Multiple visual properties are modifiable in real-time. While the color of each profile can be changed directly, other properties have to be accessed in the menu for individual settings. Line-widths and line styles can be changed freely from within the menu. The number of line-styles has been limited to four on purpose to allow users a wide enough selection of parameters without overwhelming them.

#### Property scales

At the heart of VOLPES is a database of over 600 different physicochemical property scales for the 20 standard amino acids and 4 standard RNA nucleotides or components thereof (amino-acid side chains, nucleosides, nucleobases etc.). The scales were sourced from the AAindex (21,22) and complemented by additional scales obtained either from the recent literature (cited within the tool) or derived to be used in VOLPES (e.g. amino-acid charge at different pH). To catalogue this vast dataset efficiently, we have categorized the scales by using the approach originally developed by Tomii *et al.*, (25). In the case of amino-acid scales, this has resulted in the following categories: α-propensity, β-propensity, charge, composition, hydrophobicity, general physico-chemical properties, RNA-affinity and other. RNA scales were grouped by expert knowledge into three groups corresponding to composition, energy-related properties or general physico-chemical properties. Upon selection of a scale of interest in VOLPES, a detailed view is shown providing a short name for the scale, a link to the AAindex, if present in the database, the explicit values of the scale, a longer description of the scale, a literature reference and a link to the Pubmed reference database, if available. This allows users to scroll through a large number of scales and identify those that are relevant for their purposes without a need for extensive prior knowledge. Once a scale is selected, it is immediately applied to the active sequence and the plot is updated accordingly.

#### Comparison

The level of similarity between different physicochemical profiles can be quantified in several ways. A pairwise matrix of correlation coefficients between all uploaded sequences is displayed automatically once two or more profiles are present on the canvas. As default parameters, the Pearson correlation coefficient *R* is displayed in the upper triangle of the matrix and the Spearman rank correlation coefficient in the lower triangle. Both can be exchanged for each other or the coefficient of determination *R*^2^ or the root-mean-square deviation (RMSD) by selecting the respective measures in the associated settings menu. The values in the matrix are dynamically updated whenever a change that affects the correlation level is implemented.

### Library

In addition to the implementation in the form of a freely accessible web server, the JavaScript library, used to power the server frontend, is freely available as a git repository (found at github.com/BarLuk/volpini). A detailed documentation can be found in the repository as well as on the server itself. The library is built upon the popular d3.js library (26), enabling comfortable access and modifications to SVG nodes as DOM elements with a wide range of custom functionality built in to accommodate the analysis of biological sequences as a special application.

#### Easy to use

In its most basic functionality, the library requires a single function call together with an already provided dataset to produce an interactive figure. To allow for additional interactivity aimed at sampling the parameter space, the drawing function just needs to be called whenever a parameter is updated to redraw the figure.

#### Wide range of applications

The library can be used in any environment in which JavaScript can be executed and HTML can be rendered. Due to the widespread use of modern web browsers, such an environment is present on virtually any computer. Besides the obvious use as the frontend of a web server, the library has already been employed to produce interactive conference posters as well as presentation slides for scientific talks. Examples can be found in the online documentation. At last, the library can also in principle be used for publishing interactive figures in the scientific literature, enabling the readers to explore the presented data themselves. While the authors would still be able to present their data using the visualization parameters they prefer, the readers could easily vary the parameters themselves for additional insight.

### Data security and access

Except for IP access logs, no other user data are stored server-side. This explicitly also refers to the specific sequences or combinations of sequences and scales that have been processed by the tool. Access to the server is free without a need to register. The server can be accessed at volpes.univie.ac.at.

## CONCLUSION

VOLPES is a freely available online tool for visualizing the physicochemical properties of biopolymers that only requires an up-to-date web browser for access. Both protein and RNA sequences are supported, with database connectivity enabling user-friendly data access. Over 600 different physicochemical property scales of amino acids and nucleobases are available in VOLPES, with detailed descriptions for each scale, allowing the user to quickly browse through. Enabling users to interact with visualization parameters in real-time makes it possible to develop an intimate understanding of the underlying dataset. The speed of interaction with the dataset, on the other hand, opens up the possibility of a data-based, exploration-driven approach to research that puts the raw data first, allowing one to develop hypotheses based on observations made on this level.

VOLPES was launched in August 2018 and it is expected to have high visibility among bioinformaticians and experimentalists alike. Since the original launch, several thousands of pageviews have been recorded, but due to the nature of the service, the exact number of analyses performed cannot be reported. The service has been tested thoroughly by internal and external testers and remains under active support.
